# Factors influencing the implementation of person-centred care in nursing homes by practice development champions: a qualitative process evaluation of a cluster-randomised controlled trial (EPCentCare) using Normalization Process Theory

**DOI:** 10.1186/s12912-022-00963-6

**Published:** 2022-07-08

**Authors:** Christin Richter, Steffen Fleischer, Henriette Langner, Gabriele Meyer, Katrin Balzer, Sascha Köpke, Andreas Sönnichsen, Susanne Löscher, Almuth Berg

**Affiliations:** 1 Institute of Health and Nursing Science, Medical Faculty, Martin Luther University Halle-Wittenberg, Halle (Saale), Germany; 2 Nursing Research Unit, Institute for Social Medicine and Epidemiology, University of Lübeck, Lübeck, Germany; 3 Institute of Nursing Science, Faculty of Medicine and University Hospital Cologne, University of Cologne, Cologne, Germany; 4 Department of General Practice and Family Medicine, Center for Public Health, Medical University of Vienna, Vienna, Austria; 5 Institute for General Practice, Medical Faculty and University Hospital, Heinrich Heine University Düsseldorf, Düsseldorf, Germany

**Keywords:** Nursing homes, Dementia, Person-centred care, Behavioural and psychological symptoms of dementia, Process evaluation

## Abstract

**Background:**

Person-centred care (PCC) has been suggested as the preferred model of dementia care in all settings. The EPCentCare study showed that an adapted PCC approach was difficult to implement and had no effect on prescription of antipsychotics in nursing home residents in Germany.

This paper reports the qualitative process evaluation to identify facilitators and barriers of the implementation of PCC in German nursing homes from the perspective of participating practice development champions.

**Methods:**

Five individual and 14 group interviews were conducted with 66 participants (staff and managers) from 18 nursing homes. The analysis was based on inductive coding to identify factors influencing the PCC implementation process. Identified factors were systematised and structured by mapping them to the four constructs (coherence, cognitive participation, collective action, reflexive monitoring) of the Normalization Process Theory (NPT) as a framework that explains implementation processes.

**Results:**

Facilitating implementation factors included among others broadening of the care perspective (coherence), tolerance development within the care team regarding challenging behaviour (cognitive participation), testing new approaches to solutions as a multi-professional team (collective action), and perception of effects of PCC measures (reflexive monitoring). Among the facilitating factors reported in all the NPT constructs, thus affecting the entire implementation process, were the involvement of relatives, multi-professional teamwork and effective collaboration with physicians.

Barriers implied uncertainties about the implementation and expectation of a higher workload (coherence), concerns about the feasibility of PCC implementation in terms of human resources (cognitive participation), lack of a person-centred attitude by colleagues or the institution (collective action), and doubts about the effects of PCC (reflexive monitoring). Barriers influencing the entire implementation process comprised insufficient time resources, lack of support, lack of involvement of the multi-professional team, and difficulties regarding communication with the attending physicians.

**Conclusions:**

The findings provide a comprehensive and detailed overview of facilitators and barriers structured along the implementation process. Thus, our findings may assist both researchers and clinicians to develop and reflect more efficiently on PCC implementation processes in nursing homes.

**Trial registration:**

ClinicalTrials.gov identifier: NCT02295462; November 20, 2014.

**Supplementary Information:**

The online version contains supplementary material available at 10.1186/s12912-022-00963-6.

## Background

According to an action plan presented by the World Health Organization an integrated, evidence-based, person-centred care (PCC) is required in all settings where people with dementia live [1]. PCC is a holistic treatment approach designed to maintain the quality of life of people with dementia and reduce challenging behaviour [2]. Based on the PCC concept by Kitwood, defining characteristics are to acknowledge the personhood of each individual in all aspects of care, to personalise care and environment, to interpret behaviour from the viewpoint of the person with dementia, and to prioritise the relationship as much as the care tasks [3].

PCC is widely spread in dementia guidelines [4, 5], and long-term care facilities are trying to implement PCC to enhance the quality of care [6]. However, implementation of PCC into practice raises challenges and depends upon patient populations and healthcare contexts [7]. Few studies describe facilitators and barriers of PCC implementation in nursing home contexts. From the perspectives of nursing home staff (managers, nurses, and care staff) and resident families, the PerCEN trial identified factors that enabled and inhibited the implementation of PCC [8]. Findings suggest that managerial leadership and support, staff and family knowledge, understanding and acceptance, and staff’s capacity to embrace change are essential when instituting PCC [8]. Other studies [9, 10] explored influencing factors to the practice of PCC from the perspective of care assistants and aged care workers (who attend more to activities of daily living of the residents and less to clinical issues than nurses). Insufficient time, limited staffing resources, and residents’ dementia behaviours are key barriers to providing PCC, while teamwork acts as facilitator. Aged care workers seem to have a reasonable but incomplete understanding of PCC in the context of their role [9, 10]. A study with a sample of researchers involved in PCC research projects described their perspectives on PCC implementation in different healthcare contexts [7]. With regard to the nursing home setting, staff culture (focussing on speech not on alternative communication aids) and workload (time needed to talk to a person who cannot speak) were reported as barriers. Multidisciplinary team meetings for PCC, PCC education, and interest in PCC were cited as facilitators.

Quite a few systematic reviews (e.g. [2, 11])  have summarised evidence on the effectiveness of PCC on people with dementia. However, results were inconsistent depending on different types of interventions and different psychosocial outcomes. The meta-analysis by Fossey and colleagues [12] indicated that person-centred training interventions for nursing home staff revealed significant benefits in improving agitation and reducing the use of antipsychotics in people with dementia. One of the analysed intervention manuals is “The Focussed Intervention of Training for Staff” [13, 14], a ten-month PCC training package for professionals working with nursing homes to achieve care improvement. The intervention was evaluated in a cluster-randomised controlled trial in the United Kingdom (UK) [13] and demonstrated a clinically relevant reduction in use of antipsychotic medication. Although there is a partial decline in the prescription rates, antipsychotics continue to be prescribed frequently in order to control behavioural and psychological symptoms of dementia (BPSD). However, they should only be used as a last resort and discontinued within three months, while non-pharmacological interventions individually tailored to the person with dementia should be the first treatment option for BPSD [5].

Thus, this promising PCC approach was adapted to German conditions by our group and evaluated in a cluster-randomised trial to examine whether this approach would result in a reduction of antipsychotic prescriptions in German nursing homes (EPCentCare study) [15, 16].

### The EPCentCare study

The EPCentCare study (ClinicalTrials.gov NCT02295462) was designed as a two-armed cluster-randomised controlled trial over 12 month. A synopsis of the study is presented below; the detailed description can be found in the study protocol [15] and the publication on the effectiveness study [16].

Overall, 37 nursing homes with *n* = 1,153 residents (intervention group: *n* = 493; control group: *n* = 660) in East, North and West Germany participated in the trial. The mean age in both groups was around 84 years and the proportion of women was around 73%.

Both study groups received medication reviews (*n* = 1,610) for all residents with an ongoing antipsychotic prescription providing feedback to the prescribing physician (in terms of optimised usual care, since medication reviews are not systematically implemented in Germany). Additionally, all of these physicians were offered access to two hours of further medical training. The control group received no further intervention other than the optimised usual care. The intervention group received an intervention programme based on the study by Fossey et al. [13].

For nursing homes in the intervention group (*n* = 18), selected staff (*n* = 90) were trained and instructed to work as *Expert for PCC for Older People (EPA)*. Table 1 displays the tasks, which were carried out by these practice development champions. At the median, one expert was responsible for five participating residents. In accordance with the programme by Fossey et al. [13, 14], the training programme for the EPAs included (i) an initial two-day workshop on PCC, and (ii) continuous in-house support during the 12-month intervention period by a study nurse specialised in dementia and PCC.

**Table 1:** Expert for Person-Centred Care for Older People (EPA) specification

*Preconditions:*
➢ Registered nurse or related profession (occupational therapy, social care) ➢ At least a 75% part-time position in the nursing home
*Tasks and responsibilities of the EPA:*
➢ Identifying organisational needs for change within the institution and opportunities for developing specific aspects of care ➢ Planning and supporting the implementation of person-centred care in the nursing facility (promotion of person-centred activities and interactions) ➢ Initiating discussions with colleagues about person-centred care activities for specific residents ➢ Discussion with colleagues about the medication review or contacting the prescribing physician ➢ Advice, guidance and support of colleagues as facilitator and mentor, and primary contact person for colleagues, relatives, and physicians

Nine initial PCC workshops (three workshops per study centre) were carried out with following content:

- Introduction to the EPCentCare study (45 min)
- Information on reducing antipsychotics in people with BPSD in favour of non-pharmacological treatment: guideline-based recommendations [17] (90 min)
- PCC on resident level: PCC according to Kitwood [3, 18], recognising individual residents’ needs and reasons for behaviour [14], dealing with challenging behaviour [19, 20], and reflection of case examples (270 min)
- PCC on institutional level: assessing, supporting and managing development of PCC in a nursing home [14, 17] (180 min)
- Overview of the supervision/support programme [13] (60 min)

In addition, staff (*n* = 295) in 17 intervention group nursing homes attended an information session about the EPCentCare study (60 min) three weeks (median) after the initial PCC workshop. In one cluster, implementation of the session was not feasible due to staffing shortage and time constraints. Here, a written presentation of the study and the role of the EPAs was provided.

The continuous in-house support for the EPAs was carried out individually and/or in groups (duration of support: 11 months). At average (median) 0,6 in-house supervision meetings with EPAs were carried out in each intervention nursing home per month during this period, i.e. more than one meeting each second month, complemented by regular telephone or e-mail contacts (median eight contacts per month) [16]. The support programme promoted a broad spectrum of (i) professional nursing expertise, (ii) competencies for participation in medical therapy, and (iii) competencies to support the implementation of PCC. These included, for example, (i) nursing interventions for people with dementia, communication strategies and dealing with challenging behaviour; (ii) administration and consequences of the reduction in antipsychotics as well as cooperation with prescribing physicians; (iii) reflection of the institutional structures and conditions, dealing with institutional barriers, limited staff and time capacities or with barriers in the team.

The adapted PCC approach revealed no benefit [16]. The proportion of residents with at least one antipsychotic medication changed after 12 months from 44.6% to 44.8% in the intervention group and from 39.8 to 33.3% in the control group. After 12 months, the difference in the prevalence was 11.4% between the intervention and control group (95% confidence interval: 0.9–21.9; *P* = 0.033).

Alongside the intervention effectiveness data, we conducted a process evaluation as recommended by the UK Medical Research Council [21, 22] to systematically obtain information on the achieved implementation of the intervention components and the contextual factors influencing implementation. As a result, the quantitative process evaluation revealed that the PCC approach was not implemented to the desired extent [16]. In some nursing homes, contextual factors such as staff and time constraints as well as working conditions impeded the EPAs in consistently fulfilling their role as disseminators. (Further details about the quantitative process evaluation with effects on intermediate outcomes have been presented in the publication reporting the effectiveness study [16]).

### Study aim

The current literature on influencing factors of PCC implementation in nursing homes does not consider the perspective of practice development champions as disseminators of the PCC knowledge among work teams. A systematic review on contextual factors influencing complex intervention research processes in nursing homes [23] revealed that staff prioritised habitual ways of working instead of novel research activities when time was limited. Likewise, sustained engagement was unlikely if staff could not identify the meaning behind the new implementation activities. On the other hand, clarity of roles, a supportive management culture, and shared understanding of the purpose of practice change within the team proved to be drivers for success [23].

Thus, to gain in-depth information and a comprehensive understanding about the complexity of the PCC implementation within the EPCentCare study, we conducted a qualitative process evaluation. Our objective was to identify barriers and facilitators of the implementation of PCC in German nursing homes from the perspective of participating practice development champions.

## Methods

### Study design

A qualitative design was used, in which analysed interview data were structured by means of the Normalization Process Theory (NPT) [24, 25] in order to gain insight into the PCC implementation process. The NPT is increasingly applied for explanation of implementation processes of complex healthcare interventions [26].

Ethical approval was obtained from the ethics committee in each study centre and written informed consent was obtained from all participants.

### Participants

After the study was completed (October 2016), all the nursing homes in the EPCentCare intervention group (*n* = 18) were asked to participate in the qualitative process evaluation. Eight nursing homes were non-profit, seven private, two church-run and one public.

The facilities varied in size with beds for up to 180 residents, the smallest having only 19 beds. The mean number was 99 (± 45) beds. The mean proportion of residents with dementia was 55.5%.

The mean age of the involved EPAs (*n* = 90) was 40 years (SD = 12); 84% were women. The majority (73%) were fully-licensed nurses (three-year vocational training); other professions covered occupational therapy and social care (both with 5%). The mean duration of professional experience was 12 years (SD = 10). The EPAs were asked to take part in a qualitative interview (group or individual interview) and 61 EPAs (68%) from *n* = 18 nursing homes participated.

### Data collection

Fourteen group interviews (*n* = 6 each in East and West Germany, *n* = 2 in North Germany) and 5 individual interviews (North Germany) were conducted on-site in the respective nursing home. The group interviews consisted of *n* = 61 participants, including five nursing managers. They lasted between 14 and 35 min (mean 24 min); individual interviews lasted between 16 and 60 min (mean 37 min). Two members of the EPCentCare study team from the associated study centre each moderated the group interviews; one person conducted the individual interviews. The interviewers were not involved in the EPA support programme.

The interviews were conducted using a semi-structured interview guide that reflected the framing themes of the MRC guidance for process evaluation [21]: context, implementation and mechanism of impact. The interviews focussed on the most important uncertainties posed by the intervention in this way improving understanding of its implementation. The interview guide covered the following key areas:

- (Facilitating and hindering) experiences of working as an EPA,
- Outcomes of PCC and changes in the care for nursing home residents,
- Reflection on the support programme.

### Data management and analysis

All group and individual interviews were audio-recorded, transcribed verbatim and anonymised.

Data analysis started after completion of all interviews in the intervention group nursing homes. The data material from both the group and individual interviews was analysed line-by-line using inductive coding to identify factors influencing the process of PCC implementation. After that, the inductively generated influencing factors were mapped to the constructs of the NPT and their specific components [25] as a conceptual framework that explains implementation processes. NPT consists of four constructs that represent different kinds of work around implementing a new practice:

1. Coherence (= making sense of the intervention),
2. Cognitive participation (= investing in the intervention),
3. Collective action (= delivering the intervention), and
4. Reflexive monitoring (= appraising the effects of the implementation).

**Fig. 1:**
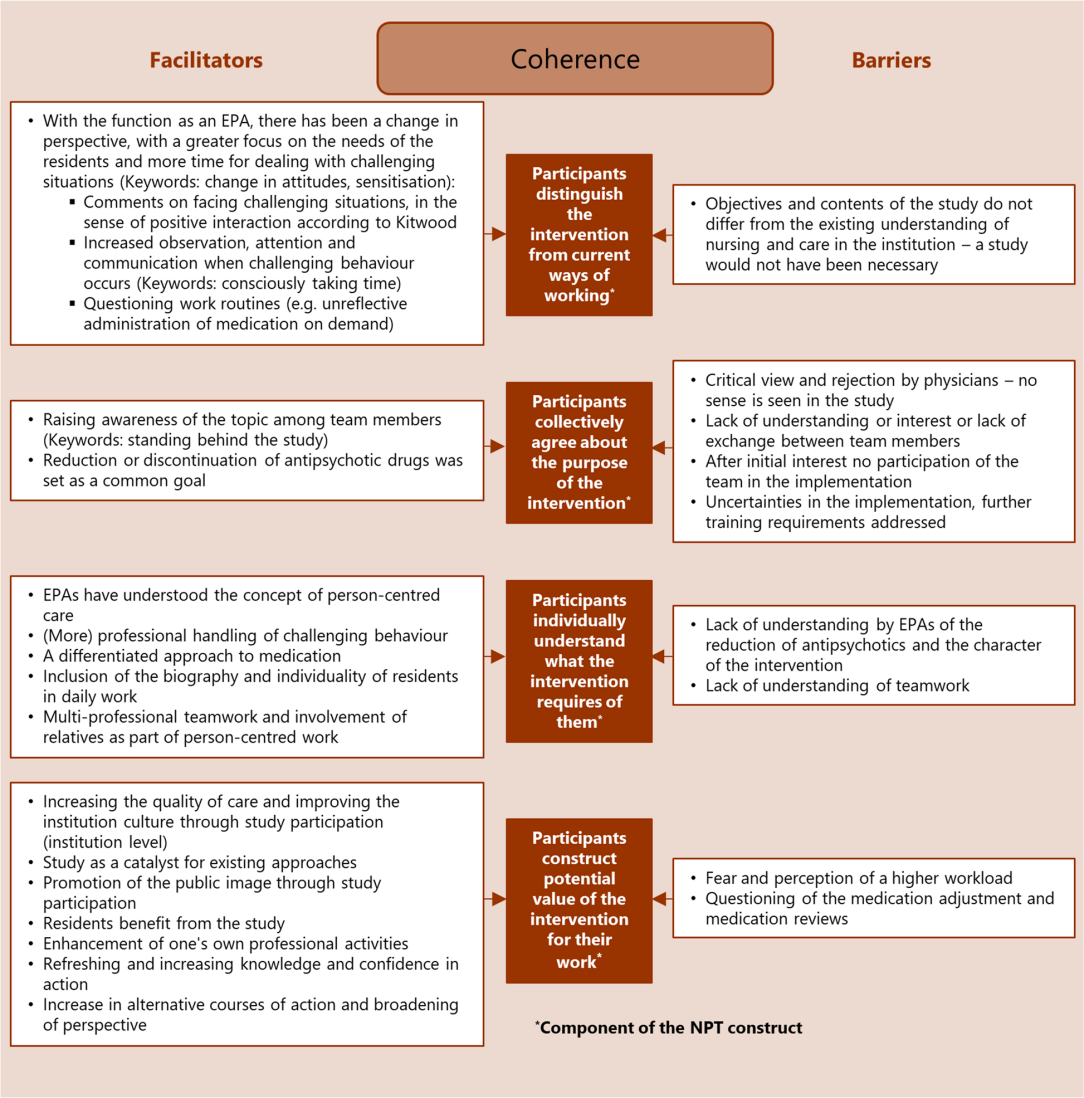
Making sense of PCC: influencing factors (facilitators and barriers) mapped to the four components of the NPT construct “coherence”

**Fig. 2:**
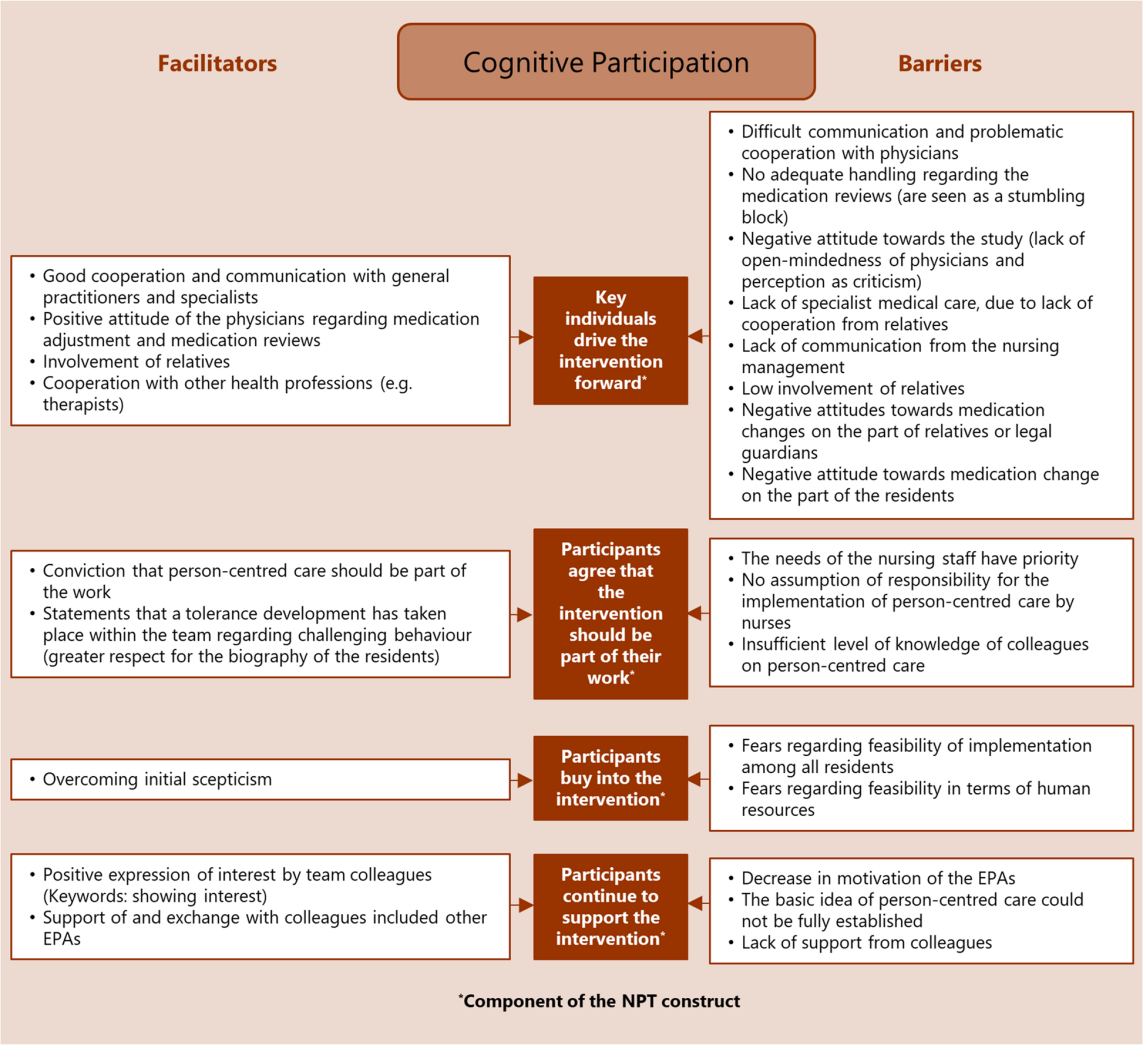
Investment in PCC: influencing factors (facilitators and barriers) mapped to the four components of the NPT construct “cognitive participation”

**Fig. 3:**
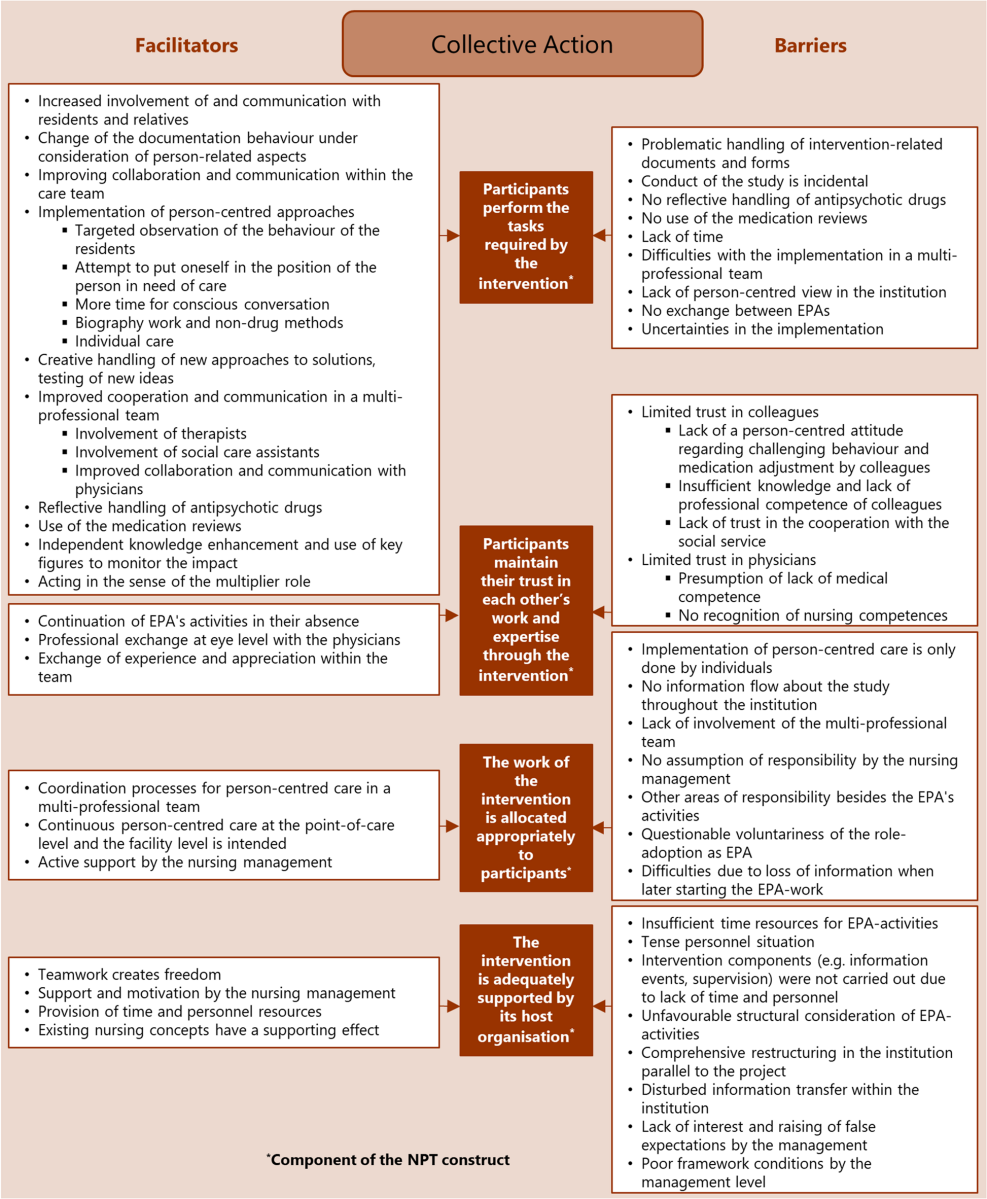
Implementing PCC: influencing factors (facilitators and barriers) mapped to the four components of the NPT construct “collective action”

**Fig. 4:**
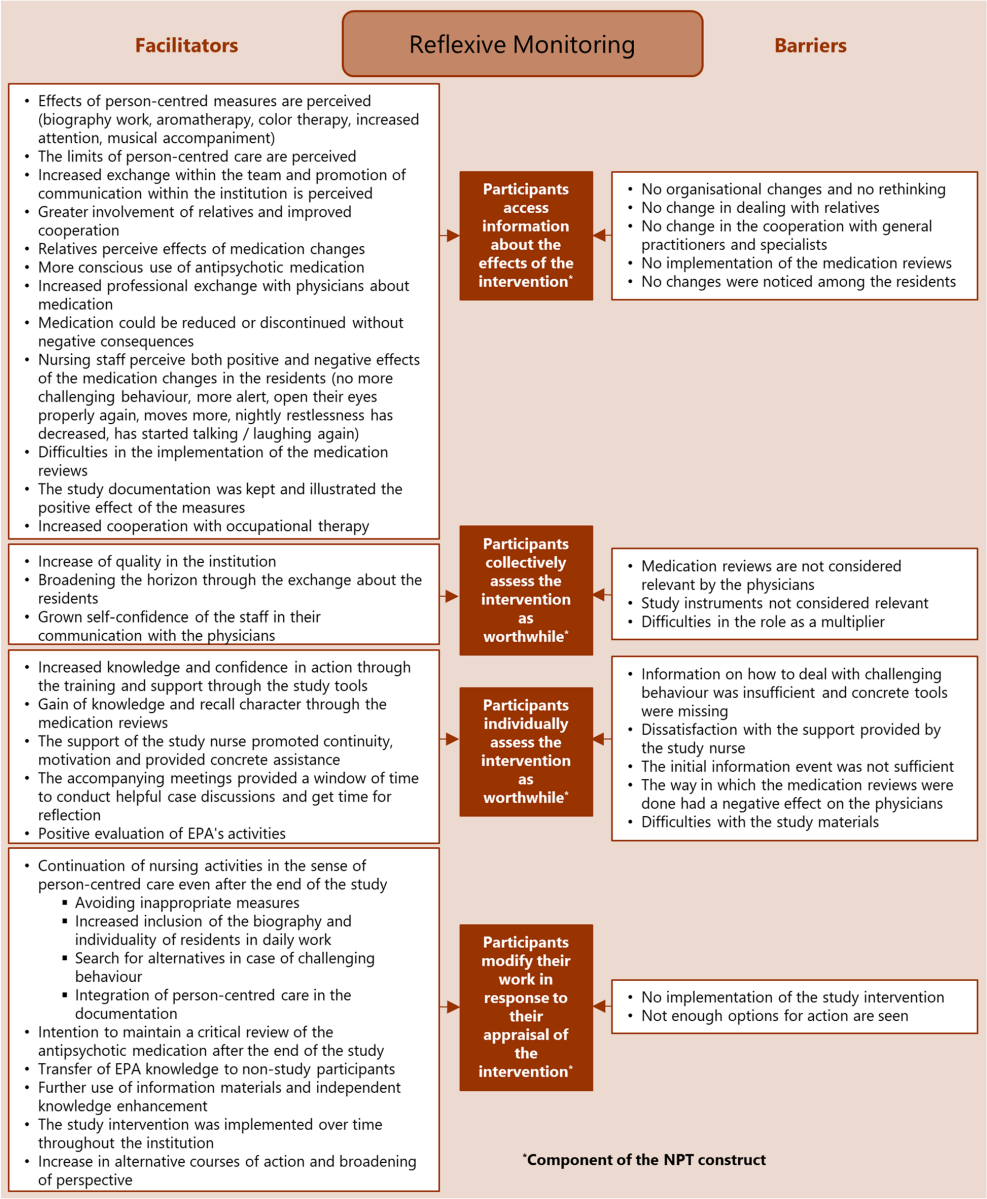
Appraising PCC: influencing factors (facilitators and barriers) mapped to the four components of the NPT construct “reflexive monitoring”

Each NPT construct consists of four components (see Figs. 1,2,3,4: brown boxes). The mapping was conducted to systematise and structure the factors identified during the implementation process. Factors that represent a negative influence on the PCC implementation process were assigned to the relevant NPT component as barriers and those with a positive influence as facilitators. A matrix with the mapping definitions can be found in Additional file 1.

### Trustworthiness

Trustworthiness of this study is based on the four criteria described by Lincoln and Guba [27]: credibility, confirmability, dependability, and transferability.

Each interviewee participated voluntarily; the first interview served as a pre-test for the interview guide. The interviewers, who had no managerial relation with participants, were also involved in processing of the cluster-randomised controlled trial, facilitating trust and understanding between researchers and members of the setting. This ensured the credibility of the interviews. Confirmability was enhanced through independent coding and mapping by two researchers (CR, HL); using MAXQDA software (www.maxqda.com). Dependability was addressed by using a semi-structured interview guide to ensure consistency as well as close reflection and discussion of the findings through involvement of a third person (AB) in regular data workshops. To achieve transferability, we provide further references to the EPCentCare study and additional files of the data analysis process that allow readers to assess applicability of the presented findings in other contexts.

The qualitative process evaluation is reported according to the Standards for Reporting Qualitative Research (SRQR) [28].

## Results

The analysis resulted in the identification of numerous factors that influence the implementation of PCC in nursing homes by practice development champions. The factors were assigned as barriers and facilitators to the four NPT constructs and their components in order to structure them in the course of the implementation process. The NPT constructs are summarised below; all identified influencing facilitators and barriers together with the assigned respective NPT construct are displayed in the corresponding Figs. 1,2,3,4.

Additional grounding quotations to the ones mentioned in the summarised results below are presented in Additional file 2. (Each quotation is addressed by its corresponding code — the letter corresponds with the study region: E = East Germany, N = North Germany, W = West Germany).

### Coherence: making sense of PCC

‘Coherence’ as sense-making work (described by four components [25]; see Fig. 1: brown boxes) focuses on processes that promote or inhibit the coherence of PCC among the EPAs, driven by investments of meaning made by the participants.

Supporting influences comprised that the participants were sensitised to the topic and the professional handling of challenging behaviour, experienced a broadening of their perspective, and learned new skills or refreshed existing ones.

*“The all-round view, i.e. to the resident, was thus reawakened. One pays more attention to small peculiarities, which would otherwise have been commonplace, but which have now come to the fore again in this study.” (E18-28)*

While participants understood the concept of PCC, some revealed uncertainties about the implementation, expected a higher workload, and experienced no participation of the care team or a lack of interest.

“Not everyone had such an open ear for it, I must say. Many found it unnecessary.” (E12-39)

### Cognitive participation: investment in PCC

‘Cognitive participation’ as relational work (described by four components [25]; see Fig. 2: brown boxes) focuses on processes that promote or inhibit legitimation of PCC, driven by investments of commitment made by the participants.

Some EPAs perceived a tolerance development within the care team regarding challenging behaviour and a greater respect for the biography of the residents. The conviction prevailed that PCC should be part of the work.

*“And we would have preferred the [resident] to sit quietly and not disturb us. But then I realise that this has also changed in the team and that it is tolerated. And yes, somehow different standards have developed. […] And in the case discussions we had, it actually grew.” (N16-8)*

However, concerns were expressed about the feasibility of PCC implementation among all residents and in terms of human resources.

“But then it was always the same: ‘How are we going to do that? We don’t have that many staff.’” (E11-30)

Lack of support from colleagues and nursing management, and problematic communication with physicians about PCC approaches instead of antipsychotic medication were mentioned as barriers.

*“I think that was the hardest part of the whole thing, communicating that to the doctors or telling the doctors, we would like to try that now. So there was only good or bad, black or white.” (E14-4)*

### Collective action: implementing PCC

‘Collective action’ as operational work (described by four components [25]; see Fig. 3: brown boxes) focuses on processes that promote or inhibit the enacting of PCC; driven by investments of effort made by the participants.

The EPAs appreciated the increasing importance that PCC had acquired, with active support by the nursing management playing an important role in implementing PCC.

*“And what I also find so important is that human things like addressing, closeness, caressing someone suddenly took on a meaning that could be written down. I was pleased when we included this in the documentation for the first time as person-centred care, because it is not self-evident. It gets lost in everyday work: standing still, waving to someone, smiling at someone, giving space to someone.” (W18-12)*

They reported a creative handling of new approaches to solutions, and coordination processes for PCC in a multi-professional team.

In contrast, lack of support and lack of a person-centred attitude by colleagues or the institution had a negative impact. Then the implementation of PCC remained only at the level of individual EPAs.

*“The ones who are still trying are really sitting here. [...] But if you don’t have any support, then you think you’re running into a wall. And then you think you are out of place at some point.” (E17-67)*

### Reflexive monitoring: appraising PCC

‘Reflexive monitoring’ as appraisal work (described by four components [25]; see Fig. 4: brown boxes) focuses on processes that promote or inhibit comprehension of the effects of PCC, driven by investments in appraisal made by the participants.

Implementation of PCC was strengthened when EPAs perceived direct effects on residents.

*“I enjoyed seeing the successes. That you really saw when you really took the time for the residents and used it intensively, that [...] they really became calmer [...] and that you give people a lot of things – in that moment.” (N12-51)*

The EPAs also reported broadening their horizon through the exchange about the residents and an increased self-confidence of the staff in their communication with the physicians.

Rethinking became difficult in case of any doubts about the effects of PCC, no changes were noticed among the residents or not enough options for action were seen.

*“Well, the question is rather whether it is enough if I send someone to look after them for an hour once a day? And the challenging behaviour is gone in that hour, but not in the remaining twenty-three hours. Is that so effective? [...] Sometimes you just don’t have the possibilities.” (E19-55)*

### Across all NPT constructs: factors affecting the entire implementation process

Facilitating factors reported in all the NPT constructs (see Figs. 1,2,3,4), thus affecting the entire implementation process, were the involvement of relatives as part of person-centred work, multi-professional teamwork and effective collaboration and communication with physicians.

Barriers influencing the entire implementation process (see Figs. 1,2,3,4) were insufficient time resources for the EPA-activities, lack of support from colleagues and the nursing management, lack of involvement of the multi-professional team, and difficulties regarding communication and cooperation with the attending physicians.

## Discussion

This qualitative process evaluation within the EPCentCare study explored the influencing factors of the implementation of PCC in German nursing homes from the perspective of participating practice development champions. We applied the NPT as a conceptual framework to structure the identified facilitators and barriers in the course of the implementation process.

Facilitating factors ranged from a broadening of the care perspective (coherence) and tolerance development regarding challenging behaviour (cognitive participation) to testing new approaches (collective action) and the perception of effects of PCC measures (reflexive monitoring). Barriers ranged from expectations of a higher workload (coherence) and concerns about the feasibility (cognitive participation) to the lack of person-centred attitudes (collective action) and doubts about the PCC effects (reflexive monitoring).

Application of the NPT not only enabled a systematic conceptualisation of the numerous influencing factors, but also guided the identification of facilitators and barriers that affect the entire implementation process, such as effective collaboration with physicians versus insufficient time resources or lack of management support.

Our findings are in line with the presented studies on facilitators and barriers of PCC implementation in nursing home contexts [7–10] and confirm them from the perspective of practice development champions. The findings are also consistent with other recently published papers on implementation processes in nursing homes, such as a systematic review specifically focussing on barriers and facilitators influencing the implementation of complex interventions targeting neuropsychiatric symptoms and psychotropic drug use in long-term care [29]. This review demonstrated that management support, support of champions, communication and coordination between disciplines, sufficient resources, and an ‘openness to change’-culture could be facilitators to implementation, while barriers were mostly related to unstable organisations, such as perceived work and time pressures.

A Norwegian cross-sectional survey [30] exploring the association between PCC and organisational factors and staff characteristics in nursing homes revealed that higher levels of PCC were associated with a lower level of quantitative demands (e.g. to work overtime or at a rapid pace) and role conflict (e.g. incompatible requests from two or more people). Higher levels of PCC were associated with a higher level of perception of mastery (e.g. to be content with the quality of the work), empowering leadership, innovative climate and perception of group work.

Based on qualitative focus groups, van Teunenbroek et al. [31] constructed a framework explaining the relationships between barriers towards achieving change in management of neuropsychiatric symptoms in nursing homes. Among other themes of barriers, ‘suboptimal communication’ and ‘inadequate (multidisciplinary) collaboration’ may cause ‘differences in perception’, which in turn can lead to ‘disorganisation of processes’ [31]. Since the principle of free choice of physicians exists in Germany, there is a large number of local outpatient physicians treating residents in a single nursing home. Consequently, the nurse–physician communication was more challenging in our study.

The present findings provide a first systematic and comprehensive overview of the influencing factors of PCC implementation in nursing homes from the perspective of practice development champions, and thus complement the existing knowledge with the views of the PCC knowledge disseminators. Our findings may assist in developing and reflecting on PCC implementation processes in nursing homes more efficiently. When assessing factors influencing the implementation, the facilitating factors should be retained or adopted if possible, and barriers should be addressed. Therefore, solutions should first be developed for barriers preventing successful implementation. Identification of factors influencing implementation is also the prerequisite for tailored implementation strategies, which can be effective, although the effect tends to be small to moderate [32]. However, more research is needed on the most effective approaches to how determinants of healthcare professional practice should be identified and which determinants are most important to address [32].

### Strengths and limitations

Participation of a large number of practice development champions (*n* = 61/90; 68%) from all intervention group nursing homes (*n* = 18) in the interviews is a strength of this qualitative process evaluation. Nevertheless, data collection took place after completion of the cluster-randomised controlled trial, thus, data collection was retrospective and might have influenced the judgement of the process of PCC. Despite the large sample size and the large number of identified influencing factors (as presented in the figures), it cannot be ruled out that data saturation was not reached, particularly since some group interviews were rather short.

Using a conceptual framework is a clear strength of our analysis, because it enabled us to structure and explain the findings during the implementation process. We were able to link all inductively generated influencing factors to the NPT constructs and their specific components. However, the factors were mapped with the NPT taking into account the construct and component of best match. Overlapping with another construct or component can therefore not be ruled out. To prevent this, the mapping was carried out independently by two researchers and discussed in regular data workshops.

## Conclusions

This qualitative process evaluation has yielded a comprehensive insight into the influencing factors of the implementation of PCC in German nursing homes as experienced by the participating practice development champions.

Applying the NPT, facilitators and barriers could be structured and presented during the implementation process. If a PCC approach is to be implemented, it is important to consider all the constructs around the implementation of a new practice. In this respect, the identified influencing factors may guide assessments of both researchers in the designing of PCC interventions and nursing home staff in the reflection of their own implementation processes.

## Supplementary Information

**Additional file 1. **Matrix with the definitions for mapping the identified influencing factors to the NPT constructs.

**Additional file 2.** NPT constructs and components with relevant quotations reflecting the facilitators and barriers for PCC implementation.

## Data Availability

The data used during the process evaluation are available from the corresponding author on reasonable request.

## Abbreviations

EPA: Expert for PCC for Older People (i.e. practice development champion)
EPCentCare: Effect of Person-Centred Care on antipsychotic drug use in nursing homes (acronym of the study)
NPT: Normalization Process Theory
PCC: Person-centred care
UK: United Kingdom
