# Supplementary material for: Factors influencing the implementation of person-centred care in nursing homes by practice development champions: a qualitative process evaluation of a cluster-randomised controlled trial (EPCentCare) using Normalization Process Theory
Source: BMC Nurs. 2022 Jul 8;21:182. doi: 10.1186/s12912-022-00963-6 (PMC9264574; doi:10.1186/s12912-022-00963-6)
Supplement: Supplementary file 1 — Additional file 1. Matrix with the definitions for mapping the identified influencing factors to the NPT constructs. [file 12912_2022_963_MOESM1_ESM.pdf]

Additional file 1: Matrix with the definitions for mapping the identified influencing factors to the NPT constructs

NPT construct and the specific components*		Definitions for mapping<br>Meaning: Encompasses statements that …
**Coherence**	1. Participants distinguish the intervention from current ways of working	… describe perceptions of differences between own (EPA's) practice and new practice OR lack thereof
	2. Participants collectively agree about the purpose of the intervention	… relate to a shared agreement of all professions regarding aims, objectives, and intention of interventions OR lack thereof
	3. Participants individually understand what the intervention requires of them	… show EPA's understanding of the concept of PCC OR lack thereof<br>… include perception of relevant practice changes to adopt PCC OR lack thereof
	4. Participants construct potential value of the intervention for their work	… describe EPA's sense-making process to attribute value and worth to PCC for themselves OR lack thereof
**Cognitive participation**	5. Key individuals drive the intervention forward	… relate to involvement of other relevant stakeholders like nursing management, physicians, relatives, social workers etc. OR lack thereof
	6. Participants agree that the intervention should be part of their work	… demonstrate EPA's agreement to incorporate PCC into their daily practice OR lack thereof
	7. Participants buy into the intervention	… show EPA's conviction that PCC can be implemented in daily practice OR lack thereof
	8. Participants continue to support the intervention	… assume continuation of at least key aspects of PCC as sustainable in the future OR lack thereof
**Collective action**	9. Participants perform the tasks required by the intervention	… describe adapted practices according to PCC OR lack thereof
	10. Participants maintain their trust in each other's work and expertise through the intervention	… show trust in competence and knowledge of involved care professions to deliver PCC as intended OR lack thereof
	11. The work of the intervention is allocated appropriately to participants	… describe assignments by nursing home management and EPAs regarding study and intervention implementation within the nursing home OR lack thereof
	12. The intervention is adequately supported by its host organisation	… illustrate direct support EPAs are receiving by their host organisation (e.g. forwarding of information, disapproving organisational culture or climate, changes in standard operating procedures) OR lack thereof

NPT construct and the specific components[*]		Definitions for mapping<br>**Meaning: Encompasses statements that …**
**Reflexive monitoring**	13. Participants access information about the effects of the intervention	… document measures executed in accordance with the study intervention and resulting effects of intervention components OR lack thereof
	14. Participants collectively assess the intervention as worthwhile	… illustrate a positive attitude of the care team and deem the intervention as meaningful and reasonable OR lack thereof
	15. Participants individually assess the intervention as worthwhile	… illustrate a positive OR negative attitude of the EPAs towards the intervention and whether they would take up the role as an EPA again OR not
	16. Participants modify their work in response to their appraisal of the intervention	… show a reflection and change of own actions in favour of the intervention or the intention to do so in future OR lack thereof

EPA: Expert for PCC for Older People (practice development champion); NPT: Normalization Process Theory; PCC: person-centred care

[*] May CR, Finch T, Ballini L, MacFarlane A, Mair F, Murray E et al. Evaluating complex interventions and health technologies using normalization process theory: development of a simplified approach and web-enabled toolkit. BMC Health Serv Res. 2011;11:245.
