# Supplementary material for: Factors influencing the implementation of person-centred care in nursing homes by practice development champions: a qualitative process evaluation of a cluster-randomised controlled trial (EPCentCare) using Normalization Process Theory
Source: BMC Nurs. 2022 Jul 8;21:182. doi: 10.1186/s12912-022-00963-6 (PMC9264574; doi:10.1186/s12912-022-00963-6)
Supplement: Supplementary file 2 — Additional file 2. NPT constructs and components with relevant quotations reflecting the facilitators and barriers for PCC implementation. [file 12912_2022_963_MOESM2_ESM.pdf]

Additional file 2: NPT constructs and components with relevant quotations reflecting the facilitators (+) and barriers (–) for implementation of person-centred care

**Normalization Process Theory (NPT)*:** Constructs	Specific components		**Relevant quotations**
Coherence	Participants distinguish the intervention from current ways of working	+	"One become blinded by routine, after many years." (W13-91)
		–	"No change - as a result of the study - occurs because the contents and objectives of the intervention are in line with the existing understanding of care and nursing in the institution." (N13-24)
	Participants collectively agree about the purpose of the intervention	+	"And many residents come from homes or hospitals. They are already being treated with psychotropic drugs. And our goal was, so to speak, to reduce or completely eliminate that if possible." (W21-24)
		–	"Not everyone had such an open ear for it, I must say. Many found it unnecessary." (E12-39)
	Participants individually understand what the intervention requires of them	+	"The all-round view, i.e. to the resident, was thus reawakened. One pays more attention to small peculiarities, which would otherwise have been commonplace, but which have now come to the fore again in this study. [...] and how to find other possibilities instead of working directly with medication." (E18-28)
		+	"Thus the view really on the individual resident and not so the view on the daily ward routine, which should be guaranteed - but on the individual resident." (N16-44)
		–	"I can't have a say, I'm only in the social care. I have nothing to do with pills." (E17-10)
	Participants construct potential value of the intervention for their work	+	"One has refreshed a lot, learned a lot of new things. [...] In the course of time, you forget a lot and you have to remember it again and again. [...] And they [psychotropic drugs] have an incredible number of side effects and what does that do to our residents. I also find this very, very interesting and I think we have dealt with this problem very intensively after this training." (W21-20)
		–	"We actually communicate very well with the [general practitioner]. [...] And we talked to her about the problem and she also thought about possibly dealing with the study. But then it quickly became clear that it doesn't make any sense." (N10-14)

Cognitive participation	Key individuals drive the intervention forward	+	“But on the whole, I must say that it worked out well with the doctors. Nobody was there who [...] immediately raised the hands and said, ‘No, I won’t do that.’ So yes, they were open to it, but simply careful. Where there were minimal doses, it was no problem letting them taper off.” (N12-77)
		–	“I think that was the hardest part of the whole thing, communicating that to the doctors or telling the doctors, we would like to try that now. So there was only good or bad, black or white.” (E14-4)
	Participants agree that the intervention should be part of their work	+	“And we would have preferred the [resident] to sit quietly and not disturb us. But then I realise that this has also changed in the team and that it is tolerated. And yes, somehow different standards have developed. Through this study. That’s how I felt. And in the case discussions we had, it actually grew.” (N16-8)
		–	“That is also far too little time to implement it. So rather the occupational therapists or the social care assistants, they can worry about it.” (E12-15)
	Participants buy into the intervention	+	“What do you expect from us? How are we supposed to manage that? When should we manage that? Why should we do that? But you’ve heard it yourself now, it has developed positively.” (E14-76)
		–	“But then it was always the same: ‘How are we going to do that? We don’t have that many staff.’” (E11-30)
	Participants continue to support the intervention	+	“But here too, with the support of colleagues, [...] not all of them, but most [...], many things are easier for me.” (E17-96)
		–	“If you take the employees themselves, I don't think anyone was interested from the beginning. You have to say it like that. So, there was this one short training session at the beginning and after that no colleague ever talked about it again. So, to this day, I don’t think they know what we do.” (E19-8)
		–	“So, you really were such a lone wolf.” (E18-35)

Collective action	Participants' perform the tasks required by the intervention	+ "And what I also find so important is that human things like addressing, closeness, caressing someone suddenly took on a meaning that could be written down. I was pleased when we included this in the documentation for the first time as person-centred care, because it is not self-evident. It gets lost in everyday work: standing still, waving to someone, smiling at someone, giving space to someone." (W18-12)<br>+ "Also, to deal with it at all. [...] What is going on in the residents? One looks at things quite differently as EPA. At least that's how I felt. One looks at our conspicuous residents very differently. What could be changed? Why is it like this?" (E18-22)
		– "And that was what we had wanted at that time, that other employees could also be lifted to this level. That is also difficult because we were not quite so sure about many things ourselves." (W19-41)
	Participants maintain their trust in each other's work and expertise through the intervention	+ "That's what I would say to the others, that communication in the field has become more professional. That is, that you have already looked at it more specifically and thought about it together. The employees also came to us in a completely different way in terms of communication. And I think that this has also led to a better relationship between nurses and physicians, [...] in other words, recognition of the professional groups." (W19-18)
		– "If we don't all pull together as a team, it's all over anyway." (E11-34)<br>– "We couldn't talk to the physicians at all; they wouldn't listen. The neurologists always know everything better anyway." (W13-15)
	The work of the intervention is allocated appropriately to participants	+ "That one really looked, that one person was responsible for the resident in the afternoon and took care of him intensively. And that also in the early morning service. [...] Also the residents, they are already looking and have a completely different connection." (N12-53)<br>+ "The greatest help was [...] that many people pulled together – including the management of the nursing home unit. Our care manager also had our backs so that we could take our time. Even if I said in between, 'I have to retreat a bit and I have to prepare myself a bit.' Then he tried to really give the time." (N12-63)
		– "We stood relatively alone." (E17-20)<br>– "You guys go ahead, let me do my job, I'll do my job as usual." (E11-30)

	The intervention is adequately supported by its host organisation	+ “[...] with a completely different concept [dementia-specific living area] right from the start, the employees are already different there.” (E12-39)
		– “I really thought that I would get some time. Like one or two days a month. That would be quite enough.” (N17-40)<br>– “If you know the neurologist is coming and then, then at least one [of the EPAs] should be there [...] to make the rounds with him.” (N17-35)<br>– “The ones who are still trying are really sitting here. [...] But if you don’t have any support, then you think you’re running into a wall. And then you think you are out of place at some point.” (E17-67)
Reflexive monitoring	Participants access information about the effects of the intervention	+ “I enjoyed seeing the successes. That you really saw when you really took the time for the residents and used it intensively, that [...] they really became calmer [...] and that you give people a lot of things – in that moment.” (N12-51)<br>+ “It was also positive that some residents were partially taken off psychotropic drugs and they blossomed, they started laughing, which was not the case for a few weeks or months.” (W21-9)
		– “I don’t think that the rethinking, that is, saying, ‘Okay, we’re trying to get away from the drug.’ [...] I don’t think that thought has really taken root.” (E11-43)<br>– “Well, I haven’t had any impact or changes from that now.” (E19-24)
	Participants collectively assess the intervention as worthwhile	+ “The conversations really woke up the employees. So, we did a lot of case discussions and you could see how the staff really showed interest and everyone knew something about the resident. Then we put everything together and saw what was best for him. And that always worked out well.” (W18-15)
		– “And that is always the difficult part. No matter what event or training you send individual employees to, the difficult thing is that these employees, who then had the crash course, have to pass on their knowledge. [...] But it really doesn’t matter what the topic is, it is difficult for them to pass on what they have heard.” (E14-110)
	Participants individually assess the intervention as worthwhile	+ “These proposals only came from my side because I took part in this study, otherwise I would not have been able to make the proposals because I did not have the know-how. So that was very, very nice for me.” (W12-4)

		+	"I would do it again. That was very positive; I also find what came out of it very positive. [...] Definitely, because I am convinced that this was a part that was missing in this area." (W21-78)
		–	"What do I do when I discontinue that? What do I do then? And then I would really have hoped [...] that we would know then: 'We have to do this'." (N10-10)
	Participants modify their work in response to their appraisal of the intervention	+	"We're moving on. So, for us, this is a very important building block and we want to continue, definitely." (W18-60)
		+	"[...], but then also for all residents in general - not only for those who took part in this project. But it also really spread to other residents." (W17-4)
		–	"Well, the question is rather whether it is enough if I send someone to look after them for an hour once a day? And the challenging behaviour is gone in that hour, but not in the remaining twenty-three hours. Is that so effective? [...] Sometimes you just don't have the possibilities." (E19-55)

* May CR, Finch T, Ballini L, MacFarlane A, Mair F, Murray E et al. Evaluating complex interventions and health technologies using normalization process theory: development of a simplified approach and web-enabled toolkit. BMC Health Serv Res. 2011;11:245.
